# Effective Mass of Quasiparticles in Armchair Graphene Nanoribbons

**DOI:** 10.1038/s41598-019-54319-3

**Published:** 2019-11-29

**Authors:** Marcelo Macedo Fischer, Leonardo Evaristo de Sousa, Leonardo Luiz e Castro, Luiz Antonio Ribeiro, Rafael Timóteo de Sousa, Geraldo Magela e Silva, Pedro Henrique de Oliveira Neto

**Affiliations:** 1 Institute of Physics, University of Brasilia, 70.919-970 Brasilia, Brazil; 2 University of Brasília, PPG-CIMA, Campus Planaltina, 73345-010 Brasília, DF Brazil; 3 Department of Electrical Engineering, University of Brasília, CP04455, Brasília, 70919-970 Brazil

**Keywords:** Materials science, Materials for devices, Theory and computation

## Abstract

Armchair graphene nanoribbons (AGNRs) may present intrinsic semiconducting bandgaps, being of potential interest in developing new organic-based optoelectronic devices. The induction of a bandgap in AGNRs results from quantum confinement effects, which reduce charge mobility. In this sense, quasiparticles’ effective mass becomes relevant for the understanding of charge transport in these systems. In the present work, we theoretically investigate the drift of different quasiparticle species in AGNRs employing a 2D generalization of the Su-Schrieffer-Heeger Hamiltonian. Remarkably, our findings reveal that the effective mass strongly depends on the nanoribbon width and its value can reach 60 times the mass of one electron for narrow lattices. Such underlying property for quasiparticles, within the framework of gap tuning engineering in AGNRs, impact the design of their electronic devices.

## Introduction

Graphene Nanoribbons (GNRs) are quasi-one-dimensional materials in which quantum confinement may lead to the appearance of a bandgap^1–5^. Strategically, GNRs are an alternative route for graphene electronics since the latter lacks a bandgap, which represents a drawback in terms of some optoelectronic applications^6,7^. GNRs present two main types of edge geometry, named armchair and zigzag^1^. Zigzag GNRs are metallic^3,4^, whereas armchair GNRs (AGNRs) may present semiconducting properties, depending on its width *N*. By its very nature, AGNRs are commonly divided into three families, denoted *N* = 3*p*, *N* = 3*p* + 1, and *N* = 3*p* + 2, where *p* is an integer^4^. Two of these families, 3*p* and 3*p* + 1, possess an intrinsic and tunable bandgap, whereas the 3*p* + 2 family presents quasi-metallic behavior, with a relevant bandgap appearing only for small values of *p*. For this reason, AGNRs are widely used in developing new optoelectronic applications^8–13^.

Despite the promising horizon to develop new solutions for organic electronics, devices based on AGNRs still offer limited efficiency^14^. In addition to the emergence of a bandgap, the characteristic lateral confinement of charge carriers in AGNRs also induce a non-zero effective charge carrier mass, which significantly affects charge carrier mobility in these materials^15^. Such picture points to the necessity of improving the understanding of charge transport to promote advances in graphene-based technology. Charge transport in AGNRs have been experimentally studied via THz spectroscopy^2^. This technique consists of optically mobilizing charges using an ultra-fast light pulse, where the interaction between charges and the THz pulse allows for the determination of the intrinsic charge conductivity. Results indicate that quasiparticles play an important role in terms of charge transport in graphene systems^2^. From a theoretical standpoint, charge transport in graphene nanoribbons has been studied in the framework of the tight-binding model^16–25^. Such studies corroborate the findings that charge carriers in these materials are indeed quasiparticles^26,27^, with polarons and bipolarons being of particular interest due to its charged nature. Polarons are quasiparticles characterized by two intragap electronic states, spin ± $\frac{1}{2}$, and charge ± *e* associated with a local lattice distortion^28^. Bipolarons, in turn, present two narrower intragap energy levels and stronger lattice distortion than polarons, charge ± 2*e* and are spinless quasiparticles^28^. The lattice distortions produced by either quasiparticle result in the observed larger effective masses responsible for reduced charge mobility. In this sense, the interplay between the carrier’s effective mass and the different properties of AGNRs is a crucial aspect that should be understood to promote the enhancement of graphene-based devices figures of merit.

Herein, we study the drift of charge carriers in AGNRs to phenomenologically characterize their effective masses (*m*_*eff*_). By means of a 2D generalization of the Su-Schrieffer-Heeger (SSH) model^29,30^, along with a Stokes dissipation model, we numerically investigate the dynamics of polarons and bipolarons in these systems. In the scope of our approach, we determine terminal velocities and effective masses of the charge carriers for AGNRs with different widths. Our findings show that effective mass strongly depends on carrier type and ribbon width, varying up to two orders of magnitude. Importantly, different carbon-based systems (or even inorganic-based nanomaterials^31^) have different electronic structures and may present distinct responses when it comes to transport properties, in the sense their symmetry and doping level may alter these properties substantially^32^. Zigzag GNR does not present an energy gap with which polarons and bipolarons are usually associated^3,4^. In this way, they are not considered here.

## Results

**Figure 1:**
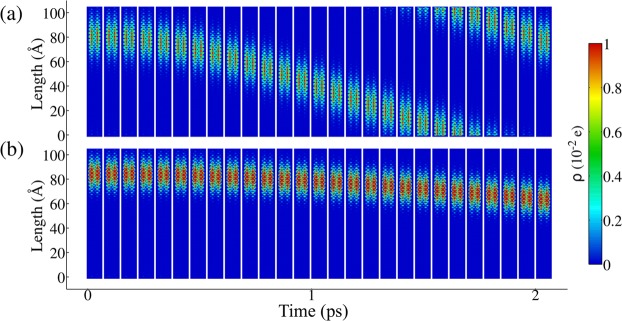
Time evolution of charge density in a 6-AGNR showing (**a**) polaron and (**b**) bipolaron motions driven by an external electric field of 1200 V/cm. Hot colors represent charge accumulation.

The quasiparticles present local lattice distortions that accumulate charge. Figure 1(a,b) depict the time evolution of charge density in 6-AGNR, where hot colors represent such charge accumulation. In Fig. 1(a), the charge density profile represents a polaron moving under the influence of an external electric field applied in the vertical direction. Similarly, Fig. 1(b) shows a bipolaron, subject to the same electric field strength. Both quasiparticles respond to the applied field. However, the polaron experiences stronger acceleration. The slower response to the electric field as presented for the bipolaron is so in spite of the its charge (2*e*), which results in twice as much force applied on bipolarons when compared to polarons. Such behavior goes to show that the extra force is not enough to balance the increased inertia of a bipolaron. Indeed, bipolarons carry along a stronger distortion of the nanoribbon lattice. Comparison between both distributions of charge density demonstrates that the polaron’s charge is distributed over 40 sites in the vertical direction, whereas the bipolarons’ charge spread over less than 30 sites. As such, the polaron is more delocalized than the bipolaron. The combination of more charge confined in shorter regions results in more significant lattice deformation, which is responsible for the increased inertia observed for bipolarons. Therefore, Fig. 1 illustrates that increased localization will take a toll on charge mobility.

**Figure 2:**
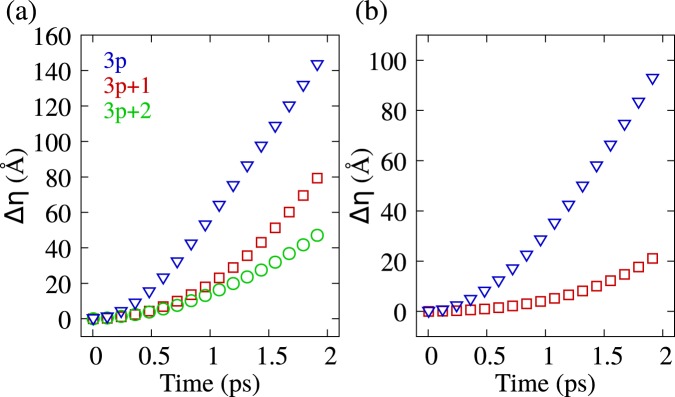
(**a**) Polaron displacement as a function of time for three different AGNRs families under the influence of different electric fields strengths. The red line represents a 4-AGNR under a field of 7220 V/cm. The green line represents a 5-AGNR for 2400 V/cm. The blue line represents a 6-AGNR 1200 V/cm. (**b**) Displacement as a function of time for two different families of bipolarons under the influence of different electric fields strengths. The red line represents a 4-AGNR under a field strength of 7220 V/cm. The blue line represents a 6x70-AGNR for 1200 V/cm.

By taking the center of the charge density distribution and registering its position, it is possible to obtain the time evolution of the charge carrier’s position in the nanoribbon. Figure 2(a) shows the polaron displacement, in the vertical direction, as a function of time. Each color represents the behavior observed in different AGNR families. Red curves refer to 4-AGNR, green to 5-AGNR, and blue to 6-AGNR. In Fig. 2(a) it can be seen that the displacement curves approach linear behavior, denoting that charge carriers reach a terminal velocity. Similar results are seen in Fig. 2(b) for bipolarons. Note that a curve representing the 3*p* + 2 family is absent in Fig. 2(b) since bipolarons are not stable in lattices belonging to this family^33^. Regarding polarons, it is worth to mention that this quasiparticles are present only in thinner AGNRs for the 3*p* + 2 family^33^, and because of that we consider as representative systems the ribbons 5-AGNR and 8-AGNR in which polarons can be formed^33^. Despite similar qualitative behavior, the two quasiparticles show different characteristic times for reaching terminal velocity. For polarons, this time corresponds roughly to 1 ps, around half of the simulation time. In the case of bipolarons, an extra 0.5 ps is necessary. A simple Stokes dissipation model is used to describe the observed carrier motion. This model considers a dissipation term proportional to the first power of the velocity: *F*_*d*_ = −*bv*. Therefore, we can obtain terminal velocity *v*_*t*_ when the dissipation term equals the force produced by the electric field *v*_*t*_ = *qE*/*b*, where *q* stands for the carrier’s charge and *E* the electric field. Under these conditions, the displacement of the charge center as a function of time (*t*) is given by

1 $$\Delta \eta (t)={v}_{t}[t-\frac{{m}_{eff}}{b}(1-{e}^{-bt/{m}_{eff}})],$$

where *m*_*eff*_ stands for the effective mass of the carrier and *b* is the drag coefficient. By fitting the displacement curves, changing the values of *m*_*eff*_ and *b*, we can evaluate both polarons’ and bipolarons’ effective masses as well as the drag coefficients. Note that meff is a fitting parameter. In this sens, we are able to evaluate both effective masses and drag parameter by using its value. Importantly, the main goal of the present work is proposing a route to evaluate the effective masses of quasi-particles in graphene nanoribbons. This quantity is fundamental to experimental approaches to the evaluation of charge mobilities, as well as the understanding of underlying properties of charge transport in graphene systems. Regarding the model used for the determination of the effective mass, as mentioned above, we chose Stoke’s model for its simplicity and its phenomenological aspect. A vital feature of this model comes from observation of terminal velocity and thus the necessity of a dissipation term.

**Figure 3:**
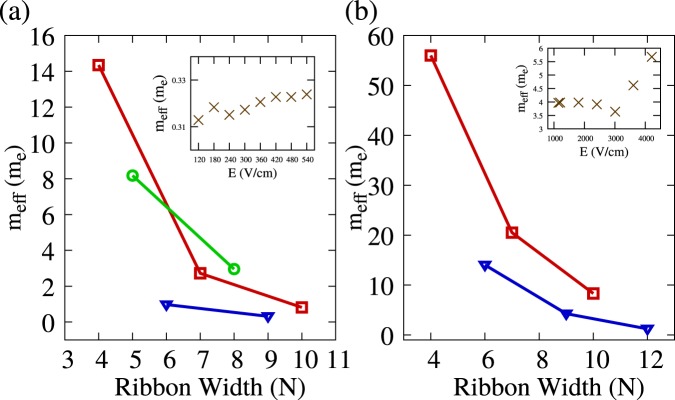
(**a**) Shows the effective mass of polarons as a function of nanoribbon width, separated into families. The red line represents 3*p* + 1 family, the green line represents 3*p* + 2 family, and the blue line represents 3*p* family. The inset depicts the polaron’s effective mass as a function of the electric field, for the 9-AGNR. (**b**) Shows the effective mass of bipolarons as a function of nanoribbon width, also separated into families. The red line represents 3*p* + 1 family and the blue line represents 3*p* family. The inset on the right illustrates the bipolaron’s effective mass as a function of the electric field, for the 9-AGNR. The values shown in this figure are obtained by using a mean value of all applied fields for polarons (panel (a)) and as well a mean value of field strengths smaller than 3000 V/cm in the bipolaron case (panel (b)).

Through the procedure described above, it is possible to understand how the ribbon width affects charge carriers inertia. Figure 3(a) shows the polaron effective mass in units of electron mass (*m*_*e*_) as a function of nanoribbon’s width. The blue, red, and green lines correspond to the 3*p*, 3*p* + 1 and 3*p* + 2 families, respectively. In all cases, the effective mass correlates inversely with the ribbon width, ranging from 0.2 to 14 *m*_*e*_. As expected, effective masses are lower for the 3*p* + 2 family, given its quasi-metallic nature. The remaining two families, however, show no clear ordering, with both curves intersecting each other. Analogous conclusions hold for bipolarons, as shown in Fig. 3(b).

In contrast to polarons, bipolaron effective masses are considerably larger, ranging from around 10 to near 60 *m*_*e*_ in the case of the 3*p* + 1 family and from 5 to 15 *m*_*e*_ in the case of the 3*p* family. These results reflect the above mentioned more considerable inertia observed in the bipolaron’s response to the electric field, responsible for the longer times needed for terminal velocity to be reached. Due to the differences in effective mass, the required electric field intensity required to move both charge carriers differ considerably. The insets of Fig. 3 show representative results of the evaluation of effective mass under different electric fields. One can note that in the case of polarons, the obtained masses are practically field independent. The same situation occurs for bipolarons until fields of around 3000 *V*/*cm*. After this critical field strength, the effective masses increase, and the fits to the model are not appropriate, indicating that the model may be not valid in high electric field regimes. This behavior takes place due to strong electron-phonon interaction in GNRs. Below a critical field strength, electrons and phonons are strongly coupled. Above this critical limit, electrons are decoupled from the lattice assuming supersonic velocities. Therefore, the lattice distortions and electron starts to move disconnectedly, and the kinematic model is not valid anymore.

**Figure 4:**
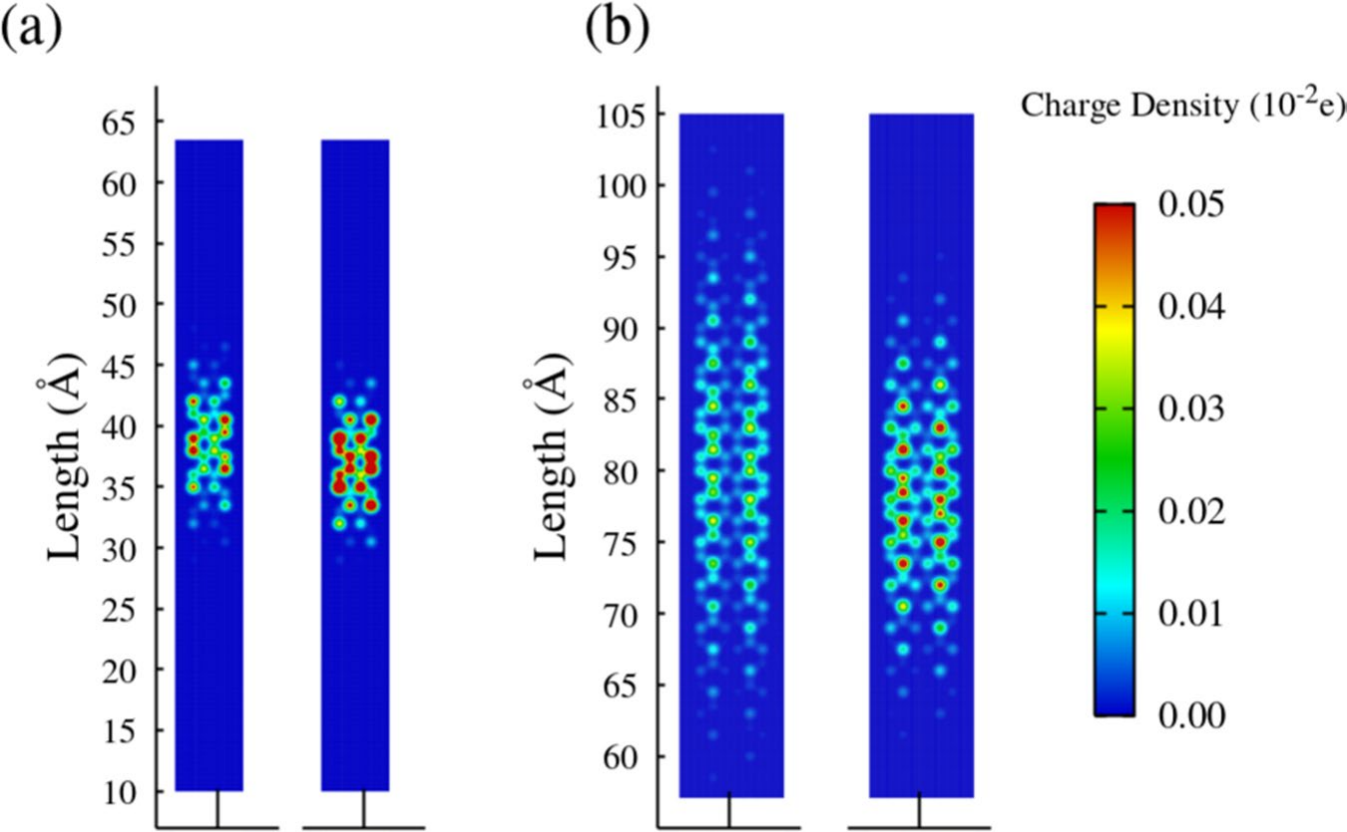
Charge density (**a**) for the 4-AGNR and (**b**) for the 6-AGNR. As the width grows, both polaron and bipolaron get more delocalized.

Finally, the interplay between effective masses and ribbon width can be understood from a microscopic perspective by analyzing the charge distribution for quasiparticles in different AGNRsy. Figure 4(a,b) show these distributions for 4-AGNR and 6-AGNR, considering a lattice containing a polaron (left panels) and a bipolaron (right panels). It is clear that as ribbon width increases, the polaron becomes more delocalized, as can be seen by the hotter colors in smaller widths. Despite increasing the ribbon width, the charge tends to concentrate laterally. The same qualitative behavior takes place for lattices containing a bipolaron. The combination of these two underlying effects for the net charge localization leads to an increase in the local lattice deformations associated with the presence of charge for narrower AGNRs. Contrarily, for wider AGNRs, the interplay of these two effects decreases the local distortions that are interacting with the charge. Moreover, in Fig. 4 it is possible to note that lattices containing a bipolaron presents a higher degree of charge localization (that are represented by the signatures in red). Bipolarons quasiparticle have a similar extension to the polaron, approximately 30 Å. Since polarons and bipolarons are composite quasiparticles in which the local lattice distortions are coupled to an additional charge, both evolve in time together during the transport of these quasiparticles. Therefore, the higher the degree of distortion more lattice energy should be transferred between neighboring sites to accomplish the polaron/bipolaron transport. Consequently, this mechanism for charge transport increases the effective mass of more localized charge carriers.

## Methodology

The model Hamiltonian employed here is given by *H* = *H*_*latt*_ + *H*_*elec*_, where the first and second terms govern the lattice and electronic degrees of freedom, respectively. By employing a harmonic approximation^30^, we treat the lattice dynamics classically. In this sense, its Hamiltonian assume the following form

2 $${H}_{latt}=\frac{1}{2}\sum _{i}\,\frac{{P}_{i}^{2}}{M}+\frac{1}{2}\sum _{\langle i,j\rangle }\,K{\eta }_{i,j}^{2},$$

where *P*_*i*_ is the momentum of the *i*-th site with mass *M*, and *K* is the force constant associated with the *σ* bond^30^.

The electronic Hamiltonian, in turn, describes the *π*-electrons dynamics according to the equation below,

3 $${H}_{elec}=-\sum _{\langle i,j\rangle ,s}\,{t}_{i,j}\{{e}^{-i\gamma \overrightarrow{A}\cdot {\hat{r}}_{i,j}}{C}_{i,s}^{\dagger }{C}_{j,s}+{e}^{i\gamma \overrightarrow{A}\cdot {\hat{r}}_{i,j}}{C}_{j,s}^{\dagger }{C}_{i,s}\}.$$

**Figure 5:**
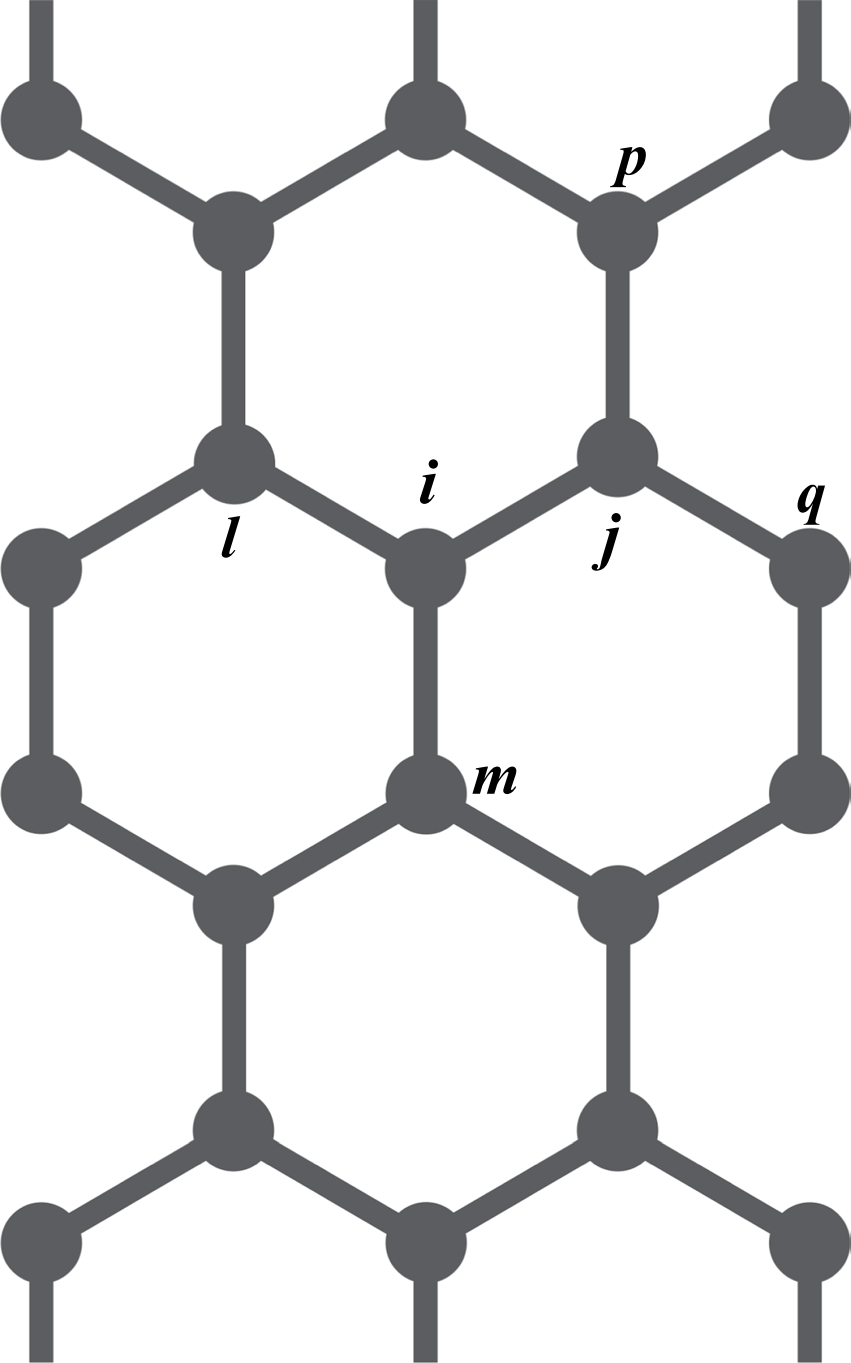
Schematic representation of the model AGNR lattice. This figure was obtained by using Gnuplot (see http://www.gnuplot.info).

The summation runs over *π*-electrons in neighboring *i* and *j* sites with spin *s* (see Fig. 5). ${C}_{i,s}^{\dagger }$ and *C*_*i*,*s*_ denote the creation and annihilation of an electron in states denoted by their subscript indices. To consider an external electric field ($\overrightarrow{E}$), we use a vector potential according to $\overrightarrow{E}(t)=-\,(1/c)\mathop{A}\limits^{}(t)$. The exponentials come from the Peierls substitution method^34^. The unit vector ${\hat{r}}_{i,j}$ points from *j* site to *i* site. Finally, the parameter *γ* in *H*_*elec*_ is defined as $\gamma \equiv \frac{ea}{\hslash c}$, where *a* is the lattice parameter, *e* the fundamental charge, and *c* the speed of light. The term *t*_*i*,*j*_ is the hopping integral, which couples the *π*-electrons to the lattice according to

4 $${t}_{i,j}={t}_{0}-\alpha {\eta }_{i,j}.$$

In Eq. 4, *α* is the electron-phonon coupling constant and *η*_*i*,*j*_ is the relative displacement of the lattice sites from their equilibrium positions.

The dynamics calculation starts from an arbitrary initial set of coordinates {*η*_*i*,*j*_}, that is necessary to solve the electronic part of our model Hamiltonian initially. As a consequence, this procedure leads to an eigenvalue-eigenvector equation for the electronic component of the system, where the eigenvalues are *E*_*k*_ and the eigenvectors are *ψ*_*k*,*s*_(*i*,*t* = 0). These quantities can be related as follows:

5 $${E}_{k}{\psi }_{k,s}(i,t=0)=-\,{t}_{i,j}{\psi }_{l,s}(j,t=0)-{t}_{i,j}{\psi }_{l,s}(j^{\prime} ,t=0)-{t}_{i,j}{\psi }_{l,s}(j^{\prime\prime} ,t=0),$$

where *i*, *j*, *j*′ and *j*″ are neighboring sites.

To solve the classical component or our model, that describes the lattice structure, we turn to the Euler-Lagrange equation^23^. From the solution of the electronic part, we evaluate the expectation value of the wave function 〈Ψ|*L*|Ψ〉. This equation leads to:

6 $$\langle L\rangle =\frac{1}{2}\sum _{i}\,\frac{{P}_{i}^{2}}{M}-\frac{1}{2}\sum _{\langle i,j\rangle }\,K{\eta }_{i,j}^{2}+\sum _{\langle i,j\rangle ,s}\,\{{t}_{i,j}{B}_{i,j}+{\rm{c}}.{\rm{c}}.\},$$

where

7 $${B}_{i,j}={\varSigma ^{\prime} }_{k,s}\,{e}^{-i\gamma \overrightarrow{A}\cdot {\hat{r}}_{i,j}}{\psi }_{k,s}^{\ast }(i,t){\psi }_{k,s}(j,t),$$

couples the electronic and lattice degrees of freedom. The primed sum means that only the occupied states are considered.

The solution of the Euler-Lagrange equation with *P*_*i*_ = 0 leads to a new set of coordinates {*η*_*i*,*j*_} that is used to recalculate the electronic Hamiltonian. This process is repeated iteratively until they reach the convergence criteria. As a result, this self-consistent procedure yields the ground state geometry that considers the interdependence between charge and lattice.

After achieving the convergence criteria, the time evolution of the initial state can be accomplished using the full Euler-Lagrange equation^23^. The time evolution of the electronic part is governed employing the time-dependent Schrödinger equation. To do so, we expand the wave function *ψ*_*k*,*s*_(*t*) in the basis of eigenstates of the electronic Hamiltonian, $\{{\phi }_{l,s}(t)\}$, at a given time *t*. Therefore, the wave function in time *t* + *dt* can be expressed as

8 $$\begin{array}{l}|{\psi }_{k,s}(t+dt)\rangle ={e}^{-\frac{i}{\hslash }{\int }_{t}^{t+dt}dt^{\prime} H(t^{\prime} )}|{\psi }_{k,s}(t)\rangle ={e}^{-\frac{i}{\hslash }H(t)dt}\sum _{l}\,|\phi {(t)}_{l,s}\rangle \langle \phi {(t)}_{l,s}|{\psi }_{k,s}(t)\rangle =\sum _{l}\,\langle \phi {(t)}_{l,s}|{\psi }_{k,s}(t)\rangle {e}^{-\frac{i}{\hslash }{\varepsilon }_{l}(t)dt}|\phi {(t)}_{l,s}\rangle ,\end{array}$$

or in terms of eigenfunctions,

9 $${\psi }_{k,s}(i,t+dt)=\sum _{l,m}\,{\phi }_{l,s}(m,t){\psi }_{k,s}(m,t){e}^{(-\frac{{\varepsilon }_{l}(t)}{\hslash }dt)}{\phi }_{l,s}(i,t),$$

where ${\varepsilon }_{l}(t)$ is the eigenenergy of ${\phi }_{l,s}(t)$.

The dynamics of the electronic structure is carried out by using Eq. 8, that is evaluated numerically and then employed to the calculation of the expectation value of a new Lagrangian^23^. The Euler-Lagrange equation leads to a Newtonian type expression that takes into account the neighboring bonds:

10 $$\begin{array}{l}{F}_{i,j}(t)=M{\ddot{y}}_{i,j}=\frac{1}{2}K\{{y}_{i,l}+{y}_{m,i}+{y}_{j,p}+{y}_{q,j}-4{B}_{i,j}\}+\frac{1}{2}\alpha \{{B}_{i,l}+{B}_{m,i}+{B}_{j,p}+{B}_{q,j}-4{B}_{i,j}+c.c.\}.\end{array}$$

The applied electric field is turned on adiabatically, to avoid numerical error, in the following scheme:

11 $$\overrightarrow{A}(t)=\{\begin{array}{ll}0 & {\rm{if}}\,t < 0,\\ -\frac{1}{2}c\overrightarrow{E}\{t-\frac{\tau }{\pi }\,\sin (\frac{\pi t}{\tau }))\} & {\rm{if}}\,0\le t < \tau ,\\ -c\overrightarrow{E}\{t-\frac{\tau }{2}\} & {\rm{if}}\,t > \tau ,\end{array}$$

where *t*_*f*_ is the total time of simulation and *τ* is the time needed until the electric field reaches its full strength. Here *τ* = 100*fs*.

To avoid edge effects, we consider periodic boundary conditions in the vertical direction of the nanoribbon, where the field is applied. Here, we use the notation NxM-AGNR, where N and M represent the number of sites on the horizontal and vertical directions of the nanoribbon, respectively. As all systems considered have *M* = 70, for the sake of simplicity, we use the notation N-AGNR. In the studied cases, *N* vary within the interval of 4–12.

## Conclusions

In summary, charge carrier dynamics in AGNRs under the influence of an external electric field were analyzed employing a 2D generalization of the SSH Hamiltonian. AGNRs with widths ranging from *N* = 4 to *N* = 12 were studied. Results point to the polaron and bipolaron formation on such systems, where these quasiparticles respond differently to the external electric field, being the inertia of bipolarons larger. Eventually, however, both quasiparticles stop accelerating under the electric fields, moving afterward with constant velocity. Making use of a Stokes dissipation model, we were able to determine the effective mass of the charge carriers for several ribbon widths. It is shown that the effective mass of these quasiparticles varies drastically depending on two aspects, the system’s width and the particular kind of quasiparticle present in the system. The effective mass for polarons presented values from 0.31 *m*_*e*_ to 14.7 *m*_*e*_. In the case of bipolarons, the effective mass had values between 4 *m*_*e*_ and 60 *m*_*e*_.
